# The potential of a hygiene-based message, preferred learning modalities, and *iLubricarte, Liberarte, Lavarte+!” or L 3 + for the prevention of HIV/STI in Peru

**DOI:** 10.21203/rs.3.rs-4889345/v1

**Published:** 2024-09-20

**Authors:** Ricky Timmons-Vendryes, Jesus Cisneros Asca, Dallas Swendeman, Alfonso Silva-Santisteban, Kelika Konda, Shahzrad Bazargan, Jesse Clark, W. Scott Comulada, Carlos Cáceres, Franceska Leon Morris

**Affiliations:** Charles R. Drew University of Medicine and Science; National University of San Marcos; at UCLA; Cayetano Heredia University; Cayetano Heredia University; Charles R. Drew University of Medicine and Science; at UCLA; at UCLA; Cayetano Heredia University; Cayetano Heredia University

**Keywords:** Hygiene, lubrication, cleansing, urination, douching, learning, intervention

## Abstract

**Background:**

Hygiene-based practices of lubrication, genital cleansing, postcoital urination, and rectal douching are common behaviors among populations at higher risk of human immunodeficiency virus (HIV)/sexually transmitted infections (STI). Yet, the role these behaviors have on HIV/STI risk has not been well elucidated, especially among transgender women (TW) and gay, bisexual, and other men who have sex with men (GBMSM). Additionally, advances in biomedical strategies have heralded a new era of HIV/AIDS prevention that may be accompanied by behavioral changes that lead to decreases in condom usage and subsequent changes to STI sequelae. Nevertheless, many people at higher risk are not benefiting equally from these options, strengthening the need for more sustainable, evidence-based methods.

**Objectives:**

This study explored the knowledge, attitudes, and behaviors of hygiene-based practices, proven preventative methods, and preferred learning methods among Peruvian TW and GBMSM.

**Methods:**

30 interviews and 50 questionnaires were conducted with TW (N=35), GBMSM (N=35), healthcare providers (N=5), and key community informants (N=5).

**Results:**

Most participants perceived hygiene-based practices to be common behaviors and a significant aspect of sexual wellbeing. Educational materials utilizing social media and hosting in-person events were also viewed favorably, with value to enhancing HIV/STI knowledge.

**Conclusions:**

Several barriers to autonomy surfaced in the data, including systemic disparities to adequate HIV/STI services, top vs. bottom social dynamics, and PrEP accessibility issues. Continued work is needed to address the barriers to the acceptability, feasibility, and potential efficacy of hygiene-based practices, biomedical/barrier strategies, and *L*_*3*_+.

## BACKGROUND

Hygiene-based and other related practices in people assigned male at birth is a forgotten area of research that may prove to be important for HIV/STI prevention [1]. Evidence supports microscopic changes that predispose people who engage in lubrication, genital cleansing, postcoital urination, and rectal douching to alterations (either increases or decreases) in one’s risk of HIV/STI transmission or acquisition –with risk varying based on the timing, type, and specific technique of the practice performed [2–8]. For all populations, hygiene-based practices are potentially modifiable behaviors that may prove to have a significant impact on one’s sexual health. Additionally, there are valid concerns that widespread uptake of pre-exposure prophylaxis (PrEP)/post-exposure prophylaxis (PEP), antiretroviral therapy (ART), and *Undetectable equals Untransmittable* (U = U) functioning as Treatment as Prevention may result in behavioral changes that lead to increases in STI incidence [9–11]. As populations adopt these new strategies of HIV/acquired immune deficiency syndrome (AIDS) prevention, some people may be less motivated to use condoms, which may lead to inattention to STI-preventative health and increased STI risk, specifically *Neisseria gonorrhea, Chlamydia trachomatis*, and syphilis [12]. Despite this possible trend, access and consistent uptake of proven HIV/STI services and treatment remain a tragic issue among TW and GBMSM of color—communities disproportionately vulnerable to HIV/STI [13, 14]. Continued support is needed to maintain and expand the achievements of HIV/AIDS-related services while also prioritizing innovative strategies that address the higher incidence of STIs for those historically left behind [15].

Understanding common behaviors and attitudes that impact sexual health is essential to inform the development of effective sexual health interventions [16]. One promising approach to HIV/STI-preventative care is to prioritize efforts on sexual health interventions that leverage mobile and social media technologies [17]. Therefore, a renewed focus on hygiene-based practices may be warranted for developing educational campaigns that utilize preferred modalities (such as social media, animated videos, websites, and printed materials) [18, 19]. Of particular interest are the attitudes and knowledge of potential benefits, harms, and safest techniques of lubrication, genital cleansing, postcoital urination, and douching. This study addresses this gap among TW and GBMSM in Lima, Peru, and surrounding districts.

## METHODS

### STUDY SETTING, RECRUITMENT PROCESS, & PARTICIPANTS

Utilizing a mixed methods research design, this formative project was an exploratory study between November 2022 and December 2023 that primarily assessed the knowledge, attitudes, and behaviors of hygiene-based practices and proven biomedical/barriers strategies of Peruvian TW and GBMSM. Preferences for learning and educational messaging modalities were also explored to inform the conceptualization of a future intervention in close collaboration with proven biomedical and barrier strategies. Participants were recruited from public health centers, initially at *Epicentro* (in the Barranco District), which holds ongoing alliances in sexual health with the Center for Interdisciplinary Research on Sexuality, AIDS, and Society (CIISSS) and UPCH. Recruitment was also conducted through other valued organizations that prioritize sexual health and LGBTQ + rights, including the *Asociación Civil de Mujeres Trans Amigas Por Siempre del Pem(in* the Callao District), *Comedor Popular Nancy de Lino* (in the Callao District), *Mecanismos de Coordinación Comunitaria Voluntades Lima Norte* (in the Independencia District), and *Mecanismos de Coordinación Comunitaria Casa Diversa* (in the La Molina District). Considering the hidden nature of our target population at times, the recruitment strategies for the interviews and questionnaires occurred exclusively through snowballing (or word-of-mouth) and the use of flyers placed in physical and online spaces of the affiliated organizations mentioned above. Additionally, the electronic messaging tool and the integration of QR codes utilizing *Qualtrics* underwent development testing and implementation during this phase. All recruitment processes were carried out following the highest ethical standards of the UPCH Institutional Review Board (IRB), as participation was individualized, voluntary, and transparent. If willing and eligible, study participants were given S/100 for their time during the interviews and S/50 for their time to complete the questionnaire.

The eligibility criteria for TW and GBMSM included **(1)** self-reported, 18 years or older adults who identified as TW or GBMSM, **(2)** ownership of a mobile smartphone (such as an iOS, Android, Windows, or Google operating system), **(3)** recent engagement of sexual activity, and **(4)** ability to participate in-person, or via phone/video call. The eligibility criteria among healthcare providers (HCP) and key community informants (KCI), who also identified as TW or GBMSM, included **(1)** self-reported, 18 years or older adult, **(2)** confirmation of being a representative or a member of an organization prioritizing the advancement or health of TW/GBMSM communities, and **(3)** ability to participate in-person, or via phone/video call.

### QUALITATIVE AND BEHAVIORAL MEASURES

A mix of TW (N = 10), GBMSM (N = 10), HCPs (N = 5), and KCIs (N = 5) were first asked to schedule and participate in a face-to-face, semi-structured interview in safe spaces of affiliated organizations. The themes used in the interviews were referenced from the Information-Motivation-Behavioral (IMB) Model, the Health Belief Model, and the core components of acceptability and feasibility [20–24]. The interviews covered several key areas, including **(1)** the personal and cultural attitudes, knowledge, and experiences of lubrication, genital cleansing, postcoital urination, and douching, **(2)** recommendations of preferred intervention modalities and learning methods, and **(3)** attitudes of intervention implementation in social, online, and clinical settings. The prepared questions were referenced as a guide, but interviews were conducted genuinely and logically to enable participants to explore topics freely. All interviews were confidential, and no identifying information was collected from any participant. All participants were also required to provide a fake name during their audio-recorded interview to refer them as. The duration of any interview session did not exceed 2 hours.

### QUANTITATIVE AND SOCIODEMOGRAPHIC MEASURES

Following a preliminary analysis of the interview transcripts, TW (N = 25) and GBMSM (N = 25) were asked to complete an online, self-completed questionnaire via *Qualtrics*. The questionnaire was referenced from the IMB Model, the Health Belief Model, the HIV Knowledge Questionnaire, and themes that emerged during the interviews [20–22, 25]. Sociodemographic information was assessed, including age, educational level, income, residency, and household faculties. Participants’ knowledge, perspective, and usability of hygiene-based practices were primarily evaluated. Experiences and knowledge regarding HIV/STI prevention and risk were collected to understand real-world patterns. Availability and acceptability of condoms and PrEP were asked to explore usage patterns. The use of mobile technologies for health-related purposes and preferences in learning methods was also investigated. All surveys were confidential, and no identifying information was collected from any participant. All participants were provided with a unique study-specific identification number. The duration of any survey session did not exceed 1 hour and 15 minutes.

### DATA ANALYSIS

First-generating descriptive statistics were employed to analyze the questionnaires and describe the characteristics of our sample. Most questions were scored on a 5-point Likert scale. Some questions were open-ended, and to further personalize one’s response, participants could choose multiple answer choices and input a typed response. Answer choices of all open-ended questions were randomized to decrease bias. All participants had the option to refuse to answer any question.

Thematic analysis techniques were employed to code and analyze the interviews. All interviews were transcribed verbatim, translated from Peruvian Spanish to English, coded, and analyzed. The coding framework developed to elicit the themes within each interview was consistently refined and systematically reviewed as it applied to the data.

## RESULTS

### CHARACTERISTICS OF THE SAMPLE

36% (18/50) of survey participants completed secondary or technical school, and 14% (7/50) completed university. 41% (20/49) of participants were unemployed, and 65% (28/43) made under S/1000 a month. 45% (21/47) lived alone, and 42% (21/50) shared bathroom facilities (such as a toilet, sink, or shower) at least most of the time with household or community members. 26% (13/50) felt it would be at least somewhat difficult to get the support they need from others (such as friends, family, and HCPs) to practice HIV-preventative methods.

“I have testimonies from boys and girls here who tell me that the nurse told me that the ampoule I am going to give you is going to hurt, but that happens because you do things you shouldn’t do”

“Dr. Elias,” identified as a HCP (nurse) and GBMSM

Many interview participants described significant, unaddressed inequities experienced among local TW and GBMSM. These disparities ranged from inattention, inactivity, stigmatization, discrimination, and victimization in many aspects of life. There is also awareness of sociopolitical disparities faced by TW that result in insecurities in health care, education, income, and nutrition. Because of these disparities and more, a few recognized that sex work was the only means of survival for most TW. Many TW expressed themselves as feeling isolated, excluded, and completely vulnerable to the disinterest of the Peruvian government and most of its citizens.

### CONDOM, PREP, & OTHER HIV/STI-RELATED PATTERNS

In the last 3 months, the majority (76%, 38/50) were at least most likely to have an adequate supply of condoms within reach, with 82% (41/50) reporting that they will at least most likely use condoms every time during sexual intercourse. 86% (42/49) of participants were aware of PrEP, but 78% (39/50) had never taken it before. The most common reasons why participants did not use PrEP included 13 participants not having access to PrEP-related services, 12 participants not knowing about the use and benefits of PrEP, and 5 participants expressing concern about potential side effects associated with PrEP use. 3 participants reported not using PrEP partly due to recommendation(s) from healthcare personnel, and 2 participants reported not using PrEP partly due to recommendation(s) from family, friends, or colleagues. Among the participants who have taken PrEP before, 55% (6/11) were at least most likely to have an adequate supply of PrEP pills.

Among those who haven’t used PrEP before, 76% (26/34) are willing to take PrEP every day if proven to reduce HIV risk by at least 90%, and 74% (26/35) are willing to take PrEP every day if it is proven to have few or no side effects. If participants were to hypothetically take PrEP every day, 60% (21/35) would expect to use condoms with the same frequency as before, with 29% (10/35) expecting to use condoms at least less frequently. 53% (18/34) would expect to have about the same number of sexual partners as before, with 21% (7/34) expecting to have at least more sexual partners.

“… there are many people who are afraid to go buy a condom at the pharmacy or the stigma that they put on us just by hearing the word “condom” because we live in a purely religious country both Catholic and Christian, evangelical and always the subject of condoms, the name condom already sounds strong. It has happened to me, I speak personally, that sometimes I tell a boy: “hey go buy a condom at the pharmacy.” And he tells me: “go buy it yourself.” So, from there I see the connotation that the same gay boys are ashamed to buy a condom in a pharmacy right?… So, I feel that there should be a campaign to remove that fear by saying condom, [because] that it’s like buying gum or going to buy a cigarette, a beer… But, I think we could do with expanding it a little more to include all these little extra things that can help, and the new forms of prevention that are also coming out: PreP, post-exposure [PEP], and all that stuff. I believe that we should give a more extensive, more comprehensive approach to everything that is prevention and not just limit ourselves to saying: “no, just use your condom, use your condom.”

“Carlota,” identified as a TW

Many interview participants reported accessibility issues with PrEP. A few expressed concerns with the side effects associated with PrEP and ART. Some questioned why the Peruvian government hasn’t sponsored public health initiatives that destigmatize condoms and demonstrate the proper techniques of condom usage with water-based lubrication. Many considered hygiene-based practices with the use of condoms and PrEP as an important strategy to reduce the transmission and acquisition of HIV/STI.

### GENITAL CLEANSING

In the last 3 months, 93% (40/43) cleansed themselves at least most of the time after intercourse. 88% (43/49) were at least most likely to have regular access to a sink or shower with running water after sex. 74% (31/42) typically cleansed themselves within 15 minutes after intercourse. 69% (29/42) cleansed for at least 2 minutes. After washing, 19% (8/43) reported genital wetness. 22 participants used antibacterial soap, 11 used bar soap, and 8 used shampoo to cleanse themselves. 41 participants cleansed to become more hygienic, 19 cleansed to increase their peace of mind, 13 cleansed to decrease their risk of contracting HIV/STI, and 9 cleansed because it was recommended to them by either a close contact or by healthcare personnel. 69% (34/49) at least knew someone who consistently cleansed their genitals after sex.

In the past 3 months, 58% (29/50) were at least most likely to use hand sanitizer, hand wipes, or other alcohol-based cleansing agents to clean themselves after sex, and 26% (13/50) sometimes used an alcohol-based cleansing agent to clean themselves after sex. 34 participants used hand wipes, and 10 used alcohol-based hand sanitizer. All participants knew at least someone who consistently used hand sanitizer, hand wipes, or other alcohol-based cleansing agents.

“We always tell men that: “wash your d**k” hahahaha… Really! We tell them in reality, sometimes jokingly, right? But usually when someone approaches me, they approach me like this and I say: “have you bathed? have you washed yourself well?” If he says yes to me and then I discover when he approaches me ready for the sexual act and I perceive “oh, just there, go take a bath, go wash.” If you want and if not bye, nothing happens.”

“Oris,” identified as a KCI and TW

Most interviewees found genital washing to be an essential aspect of both sexual well-being and one’s general bathing process. Although many learned these practices from family members and social circles, many participants questioned this behavior’s utility, efficacy, and proper techniques. A few shared their experiences using wet wipes, especially when bathing facilities were unavailable or during time constraints. For almost all, genital cleansing was an essential aspect of one’s own or their partner’s sex life.

### RECTAL DOUCHING

In the past 3 months, all participants reported douching at least once before sex. 84% (38/45) had regular access to adequate amenities/supplies (such as running water, enema solution, and enema bottle) for successful douches. 71% (29/41) douched within at least 30 minutes before sex. 39 participants douched to become more clean/hygienic, 30 participants douched to make sex more pleasurable, 23 participants douched to increase their peace of mind, and 16 participants douched due to recommendations from either a close contact or from an HCP. 8 participants douched to decrease their risk of contracting HIV/STI. 32 participants used a water-based douching solution, 10 used a water-based solution with chamomile, and 3 used a water-based solution with salt or vinegar. 96% (46/48) have never shared enema supplies with sexual partners or other associates. 86% (42/49) knew at least one person who consistently douches before sex.

In the past 3 months, 59% (27/46) participants reported douching at least most of the time after sex, with 72% (28/39) douching within at least 30 minutes after sex. 83% (34/41) douched after their most recent sexual activity.

“This is a very worrying issue, I do this type of anal washing myself, right? More hygienic when you have your sexual relations. The issue is that it harms your health, due to the issue of the PH that we have in that part, right? And well, genital washing is normal, it should be done daily, right? Er, there isn’t as much danger there as in anal washings, which harms you. I see it daily with trans girls, uh, we talk about that, but the issue is also about cleanliness, and many people call it “pumping”, it’s the term used in the community. And they demand: “I want you to pump yourself very well”, and many people demand that kind of thing from them and many times they agree to do those washings. We know that we are harming ourselves, I for my part know it, but sometimes because of the issue of looking good, for a cleanliness issue, we agree. And in trans girls, I have seen that [anal washing] is constant, every day every so often, they even don’t eat many times because they don’t [get dirty], because they have their entire stomachs clean, and they don’t get the other person dirty. That is very worrying, and to improve this, enemas are also used, but really many people due to the issue of access to the enema, it is a bit expensive to use. Mostly they do it just like that with bottles.”

“Fabio” identified as a GBMSM

Douching was a considerable part of one’s sexual hygiene or that of their partner. There was a diverse range of manifestations and justifications for douching, primarily determined by the sexual position they assumed and the accessibility of proper amenities/supplies. Some recognized their complex relationship with douching and its potential harms. A few participants shared that they significantly decreased their nutritional intake to avoid the presence of fecal remnants during planned intercourse. Some questioned why there are no resources sharing this practice’s potential harms, benefits, and proper techniques.

“They feel bad, with shame they feel dirty, and the client is upset. And they don’t want that. Once, a man got annoyed with me, I told him to go to hell, I told him: jerk, where the hell are you going? He looked at me [and said]: “is that right?” Yeah dude, what do you think? I pump myself, but it’s not my fault that something was left out there. Don’t come to me with that “wash yourself well, stupid”. And he didn’t call me anymore, and he sent me to hell. And I tell the girls: you don’t have to let yourself be dominated like that. In other words, they are very submissive, and at times I get annoyed and tell them: stop fooling around, don’t let this jerk manipulate you and dominate you. “It’s just that he gives me money, I don’t know what to do.” Turn it around [penetrate him too]. “No, mother” [they reply]. Do it -I tell them- and you’ll see that he’s not going to bother you anymore.”

“Fabio”

### LUBRICATION

In the last 3 months, 65% (32/49) of participants had access to an adequate amount of lubricant within reach at least most of the time. If participants were to have lube on hand, 82% (41/50) are at least most likely to use lube before or during intercourse. 39 participants used water-based lubricants, 16 used oil-based lubricants or Vaseline, and 9 used saliva. 72% (34/47) at least knew one person who consistently uses lubricants before or during intercourse.

“There is no facility for that, that is a limitation… a limitation that the state must remedy, we consider it important. Because condoms are good for prevention, that is true, but they are used in areas that are not lubricated because we are talking about sexual practices with trans women, right? And they are used in areas, in the rectum, an area that is not lubricated, that is in itself a little dry, it will be wet but not enough to be able to receive, to withstand penetration without harm. Lubricant saves you.”

“Lucy” identified as a KCI and TW

Many interview participants acknowledged the superiority of water-based lubrication to reduce pain and rectal damage. While most stated a preference for water-based lubricants, some reported using saliva and oil-based household products for sexual lubrication due to convenience and preferences from sexual partners. Some participants noted accessibility issues with water-based lubrication and adverse symptoms associated with saliva and oil-based lubrication.

### POSTCOITAL URINATION

In the last 3 months, 39% (19/49) of participants sometimes urinated after sex, with 53% (26/49) urinating after sex at least most of the time. 91% (43/47) typically urinated within 15 minutes after intercourse. 43 participants urinated to relieve the natural urge, 12 urinated for peace of mind, 10 urinated to decrease their risk of HIV/STI, and 9 urinated due to easy accessibility of bathroom facilities. 74% (37/50) were at least most likely to have regular access to a toilet after sex. 59% (29/49) knew of at least one person who consistently urinated after sex.

“… they informed me from some training that it was convenient to urinate afterwards because that can help eliminate some -er, let’s say- bacteria or agents that can cause diseases of the urethral canals, right? Oh, and we recommend it. Have I been specifically asked questions about it? I don’t think so. People are not very aware of this topic, and it is rather something that I have to bring up in conversation and tell them: “uh, well, remember that after having sex, as much as possible it would be good for you to urinate, right? no?’, And in that way it helps to eliminate germs, er and to the extent possible even that could occur. Er, so, that’s what I recommend, but it’s not like -according to what I remember right now- they have come to ask me: “hey should I urinate? Or how is this urination?” I don’t know if it’s so well spread or if people know it so much that they don’t ask anymore, right? It’s probably not that widespread.”

“Igor” identifies as a KCI and GBMSM

Although most interview participants performed postcoital urination, some were unsure of this practice and questioned its utility and efficacy. A few participants noted the difference in one’s tendency to urinate after sex, determined if the partner is topping (performing insertive or penetrating sex), one’s hydration status, and if they previously voided.

### KNOWLEDGE OF HIV/STI TRANSMISSION & ACQUISITION

20% (10/50) were in agreement that using enemas to clean the rectum may increase the risk of contracting HIV/STI. 54% (27/50) were in agreement that using enemas to clean the rectum did not increase the risk of contracting HIV/STI. 86% (43/50) were in agreement that microscopic tears may increase the chance of contracting HIV, and 8% (4/50) did not know. 82% (41/50) were in agreement that using a lubricant before and during sex didn’t prevent oneself from contracting HIV, and 12% (6/50) did not know. 68% (34/50) were in agreement that water-based lubricant helps prevent condom breakage, and 14% (7/50) did not know. 78% (39/50) were in agreement that Vaseline, oil-based lubricant, body lotion, or cooking oils were not the correct methods of lubrication before or during sexual intercourse. 80% (40/50) were in agreement that showering or washing one’s genitals with soap and water after sex does not prevent a person from contracting HIV, and 16% (8/50) did not know. 80% (40/50) were in agreement that urinating after sex does not prevent oneself from getting HIV, and 18% (9/50) did not know.

76% (38/50) felt it would be at least somewhat easy to stay informed about HIV prevention methods.

“ do feel that the government should have more authority in working to inform their students through the [school] curriculum, like teachers should speak about this and there should be less power of the family groups to inform because usually the family groups tend to communicate or misinform through a political agenda due to a lack of knowledge of their own, so I prefer that the person who informs them about these important things to people at this age that they need is the government or someone who has studied for 5 years for that”

“Elias,” identified as non-binary

Most interview participants possessed an accurate understanding of basic HIV/STI science, specifically regarding transmission routes, condoms, water-based lubrication, and genital cleansing. However, some participants shared misguided information concerning douching and PrEP

### MOBILE & SOCIAL MEDIA TECHNOLOGIES PATTERNS

The majority (76%, 38/50) had access to a functioning cell phone most of the time, with 92% (45/49) having access to reliable cellular internet at least most of the time. 92% (46/50) used social media at least most of the time during the day. 60% (30/50) of participants sometimes used social media for health-related purposes, with 30% (15/50) of participants using social media for health-related purposes at least most of the time. 49 participants used WhatsApp, 48 used Facebook, 37 used Instagram, 36 used TikTok, and 34 used YouTube. 38 participants used social media for health-related purposes to increase knowledge about diseases, 38 participants to receive updates on advances in healthcare, and 27 participants to exchange tips and ideas on health topics. Participants’ preferences for learning modalities included 31 participants selecting animated videos, 31 selecting virtual educational materials on social media, 28 selecting printed educational materials, 27 selecting a website, and 21 selecting reminders from healthcare personnel.

*“ will tell you that,* I *see that sometimes they [printed materials] are not so effective in the sense that people receive it, they don’t even read it, they keep it or throw it away and don’t pay attention to it, that is, there is no impact like when you see something in a social network or a live broadcast, or for example, a campaign where [there’s] like a billboard… and people approach, something that catches their attention. On the other hand, a printed leaflet, people suddenly look at it and bam, they throw it away or keep it and don’t pay attention to it, right?”*

“Manuela,” identified as a TW

Most interview participants recognized the increasing convenience and usability of mobile and social media technologies. However, some acknowledged barriers to mobile technologies, including sharing devices, limitations with rural settings, and acceptability concerns with older, conservative individuals. Some participants shared optimistic perspectives regarding the integration of a hygiene-based intervention in clinical and social settings. Some also noted flyers, brochures, and in-person workshops as potential modalities to increase the awareness of a hygiene-based message.

### POTENTIAL FUTURE OF HYGIENE-BASED BEHAVIORS AND *L3+*

16% (8/50) of participants were at least in agreement that it frustrates them to think that they will have to use lube before or during sex, wash with soap and water, and urinate after sex. 20% (10/50) were at least in agreement that they would be worried about using these practices because it would harm their health. 86% (43/50) were at least in disagreement with the statement that they would be worried if their healthcare provider found out if they were using a lubricant before/during sex, urinating after sex, and washing their genitals. 84% (42/50) were at least in agreement with the statement that their sexual partners and family would support them if they used these hygiene-based practices, and 76% (38/50) believed their healthcare provider would support them as well. 82% (41/50) of participants were at least in agreement that people in their community would be interested in learning more about hygiene-based practices. 74% (37/50) were at least in agreement that people in their community would consider using lube before or during sex, washing with soap and water, and urinating after sex.

*“So, link that to the morbidity of the relationship to the physiological usefulness of that information, right? For example, using a condom prevents you from staining yourself, pulling it and continuing, putting on another and continuing immediately. Also, the other thing, that is, linking to practical issues so that they can adapt them as their own and that, for example, for those who have premature ejaculation, a condom can help them last much longer, people are going to get hooked on that*. I *type practical things so that they can assume them as their own…”*

“Dr. Hall,” identified as a HCP (doctor) and GBMSM

Most interviewees expressed an interest in learning more about hygiene-based practices. However, many reported barriers to their ability to be well-informed about condom-based and non-condom-based methods for HIV/STI prevention. Some identified the conservative government of Peru, traditional cultural norms, inaccurate HIV/STI resources, lack of LGBTQ+ representation, and substandard values of sensitivity from healthcare providers as the most notable obstacles faced by TW and GBMSM to optimizing one’s sexual and general health.

“t has to be something constant and always renewable, that is not always the same, that is, not only to make the [trans] girls come for the simple fact that I give them condoms, or in a worship I give them the money, no, but make them come for other things, right? It can also be a space for articulation or union between the girls themselves who can come together and share–what do I know–an afternoon of movies, a talk or a conversation, like we call it a “tea for aunts;” a space where the community can come and -er- at any time they want, not only to receive condoms, lubricants, right? It stays as a community, but it is something that over time, as time goes by, begins to give different -um- prevention in different ways, but for the same community, right?”

“Maisa,” identified as a TW

## DISCUSSION

This study aimed to bring awareness to the commonality of hygiene-based practices and provide a preliminary assessment of the acceptability, feasibility, and usability of lubrication, genital cleansing, postcoital urination, and douching. A diverse range of viewpoints and experiences highlighted real-world trends and needs of local TW and GBMSM. Study data indicated the potential acceptability and feasibility of water-based lubrication and postcoital urination and washing, despite some participants questioning and a few sharing misguided information about the safety and efficacy of these practices. All survey and most interview participants voiced usability with douching, despite a majority of survey participants showcasing misinformation about douching’s potential harms. In addition, an impressive number of participants reported sexual uses of alcohol-based cleansers (such as hand wipes and hand sanitizer), oil-based household products and saliva-based lubrication, and douching with chamomile, salt, and vinegar. Given the prevalence of douching and other hygiene-based practices within our sample, we question whether past epidemiological research has underrepresented the commonality of receptive and hygiene-based practices among Peruvian TW and GBMSM or whether these findings are more reflective of sampling bias. Regardless, most participants recognized that local TW and GBMSM have been longing for more accessible and accurate information about douching, lubrication, postcoital urination and washing, and its associated risks, benefits, and safest techniques.

A fair number of testimonials emerged during the interviews highlighting sociopolitical hardships to one’s autonomy in the context of HIV/STI risk. As this study was being conducted, most TW and GBMSM did not have the opportunity to start PrEP due to widespread accessibility issues. Even among the minority who were fortunate to have access to effective methods, a few participants were stigmatized during their adherence to PrEP and condoms, expressing that they were perceived as being “unhealthy” or “unholy” by others for simply prioritizing their sexual health. Interestingly, if given the hypothetical opportunity to take PrEP in the future, most wouldn’t anticipate any changes to their current behavioral practices. Nevertheless, some did predict an increased number of sexual partners and decreased use of condoms with hypothetical PrEP use. Furthermore, some bottoms (or partners performing receiving or receptive sex) shared accounts of victimization and manipulation for the sexual, typically risky, desires of dominant, masculine tops. Although there was a diverse spectrum of femininity and masculinity among our sample, these pains were disproportionately against submissive, feminine bottoms. Some have testimonies of being forced to douche, not using condoms or water-based lubricants, abusing poppers (an inhalant used to enhance sex and relax the anal muscles), and hiding HIV/STI pharmaceutical treatment. It’s reassuring that community leaders recognize this power imbalance and actively empower submissive TW and GBMSM; however, much more discussion and action are needed to dissipate this inequality.

Regarding preferred learning modalities, multi-media deliverables are a promising area for future research and public health initiatives. Our results are in line with previous findings that show Internet-based technologies as valuable vehicles for optimizing one’s sexual health–validating the importance of low-intensity modalities. Most participants shared positive attitudes towards accurate, health-related content seen on social media. Many also recognized the utility of printed materials, in-person workshops, and other outreach events to increase awareness of a hygiene-based message. Although most participants liked the direction of the project, participants’ perspectives focused on suggestions to improve our message and implementation in the future. Recognizing the conservative nature of Peruvian society, some participants pinpointed outdated ideologies and prejudices as the main driving force for the many vulnerabilities TW and GBMSM face for equitable HIV/STI care and societal attention. Understandably, some skepticism may be present regarding the acceptability of a hygiene-based message and other new HIV/STI resources due to its novelty and the traditional nature of Peru’s culture. We are grateful that our sample shared valuable strategies to overcome these barriers and ensure a collective mission in the fight against HIV/STI. General recommendations focused on ensuring satisfactory LGBTQ + representation and advisement on all future directions of L**3**+, close collaboration with government and non-government organizations, and a prioritizing mission for the outreach, protection, and advancement of TW and GBMSM communities.

Overall, interest in these hygiene-based practices was high, and many were curious about the project’s future. These preliminary results add to an under-valued body of literature showcasing the increasing justification of a hygiene-based message for HIV/STI prevention. To our awareness, this is the first study that explored the knowledge, attitudes, and behaviors of hygiene-based practices and preferred learning modalities prioritizing TW. Given the exploratory nature of our study, we did not intend to demonstrate statistical significance from our findings. Rather, we sought to identify barriers and facilitators to inform a larger acceptability, feasibility, and efficacy study. Additional work is needed to address the identified barriers and further explore the potential of a hygiene-based message in the prevention of HIV/STI.

### LIMITATIONS

Unfortunately, our data was vulnerable to considerable bias, and we encountered several limitations in this study. Despite our best efforts, sampling bias likely occurred, decreasing the external validity of our assumptions used to derive real-world trends. Most notably, these included advertising, self-selection, and healthy-user bias. Methodological and statistical limitations were also present, including our small sample size, lack of similar research studies to reference from, the use of self-reported data, cross-cultural and translational drawbacks, and the lack of advanced quantitative analysis techniques. In addition, the investigator team was unable to retrieve particular survey data, likely due to restricted access from a free *Qualtrics* subscription. This unforeseen error limited our ability to analyze participants’ demographics, sexual orientation, accessibility of cleansing agents, frequency of douching behaviors, types of preferred cleansing agents, and more. Of note, the 1st two survey participants were unable to choose multiple answer choices for all open-ended questions. Our project manager, JCA, attentively supported these individuals in real-time and this issue was quickly resolved for the remaining 48 survey participants.

### STRENGTHS

There were notable strengths of the study. The mentorship team actively prioritized the core values of the project’s mission and a holistic refinement process using the best available, evidence-based resources and a participatory design process among the research team. Our reliance on diverse organizations and public health centers within different districts of Peru significantly enhanced the depth and variety of perspectives from our sample. The team and our sample were fortunate to have JCA as a vital contributor to the project’s highest standards of transparency, sensitivity, and respect for all involved. All participants received evidence-based HIV/AIDS educational materials, referrals to available HIV-related services, and resources on local community organizations. All interview participants were given condoms and personal water-based lubricants, and provided a safe, private space to discuss sensitive topics of sexual health that are not common in “normal” social settings in Peru. During a post-research event, we additionally shared the study results with past participants, local TW-GBMSM organizations, and community partners. All participants were also provided a PDF copy of all published articles. Lastly, the results from this study may offer indirect benefits to researchers, educators, and key community members with an additional understanding of a variety of common behaviors and attitudes that may impact sexual health and practical approaches to sharing health-related information that may prove critical for the development of important sexual health interventions.

## CONCLUSIONS

This study represents a pivotal step in investigating the role of hygiene-based practices and multi-media deliverables in promoting evidence-based HIV/STI prevention education. Overall, participants found water-based lubrication, and postcoital urination and washing to be potentially acceptable and feasible to adhere to consistently. Most participants also shared significant usability with douching. In addition, mobile messaging modalities for health-related purposes and in-person events were favorably liked among participants.

## Data Availability

The raw data were generated via *Qualtrics*. The data supporting the results are available from the corresponding author (RTV) upon request.
